# A framework for rehabilitation for older adults living with dementia

**DOI:** 10.1186/s40945-022-00134-5

**Published:** 2022-04-01

**Authors:** Julie D. Ries

**Affiliations:** Center for Optimal Aging and Physical Therapy, Marymount University, 2807 N Glebe Road, Arlington, VA 22207 USA

**Keywords:** Dementia, Rehabilitation, Physical therapy, Motor learning, Relationship, Communication

## Abstract

**Introduction & Background:**

The aging of the population assures increased prevalence of Individuals Living with Dementia (ILwD) and there will be an increased representation of this cohort requiring physical rehabilitation. If physical therapists (PTs) manage these patients as they do their age-matched, cognitively-intact peers, they will likely be unsuccessful. ILwD have unique needs related to interpersonal and pragmatic components of rehabilitation. Therapeutic nihilism (doubting the benefit of therapy) is well-documented in PTs, either because of existing biases about dementia or previous challenges in working with ILwD. Physical rehabilitation eligibility and placement decisions are often made by PTs without special training in dementia, based upon brief exposure to patients in environments not well-designed for their best functioning. This can lead to underestimation of rehabilitation potential and denial of future PT services. PTs who work with ILwD desire more practical knowledge and targeted skills. Those with more education and training have a more positive attitude and outlook related to ILwD.

**Purpose:**

The purpose of this paper is to introduce a framework for rehabilitation with ILwD equipped with pragmatic ideas to facilitate therapeutic success. The four primary components of the model are: (1) Establish a personal RELATIONSHIP, (2) Use intentional verbal and nonverbal COMMUNICATION, (3) Understand and optimize MOTOR LEARNING capabilities, and (4) Create a safe, purposeful ENVIRONMENT. Specific strategies to help PTs optimize each component are provided with supporting evidence. The model is intended to be dynamic, encouraging PTs to capitalize on the most accessible strategies within their control for a given patient and setting.

**Implications:**

This framework provides a practical resource for working with ILwD with immediate implications for facilitating therapeutic success. The model is displayed in a schematic that reminds the reader of ideas at a glance within the context of each of the components. If an appreciation for this content was among core competencies required among PTs working with ILwD, perhaps there would be significantly fewer patients written off as “uncooperative” or “unable to participate” in PT.

## Introduction & background

The World Health Organization recognizes dementia as a major cause of disability and dependency, and estimates there are ~ 50 million Individuals Living with Dementia (ILwD) worldwide [1]. The impact of dementia on mobility and gait is complex. Cognitive impairment and falls are interrelated [2] and ILwD fall more and are more likely to be hospitalized from a fall than their cognitively-intact age-matched peers [3–5]. Physical therapists (PTs) have much to offer this population, but there are biases and barriers that impact rehabilitation opportunity and success. Therapists who manage ILwD like their cognitively-intact older adult patients will probably be unsuccessful, leading to frustration, underestimation of prognosis, premature discharge, and/or ineffectual care. Therapeutic nihilism (doubting the benefit of therapy), is common in PTs and other healthcare workers [6–10]. Overt negativity, where ILwD are considered void of rehab potential or “unworthy” solely based on a dementia diagnosis has been documented [9, 11]. Healthcare systems also pose challenges and this is true internationally [7, 10, 12–15]. It is difficult to provide optimal dementia care within a biomedical model or to work within facility/administrative constraints with patients who do not conform easily. Consider Patient Case #1 Part 1 (Table 1).

**Table 1:** Patient Cases

**Patient Case #1 Part 2 (Acute Care Hospital): Knowing who and what is important (Relationship building)** *Mrs. Smith’s chart identifies her husband Stan as her next of kin. With much coaxing, the patient transitions with moderate assistance to sitting at the edge of the bed. She appears nervous.* **PT:** “I can’t wait to tell your husband, Stan, how well you are doing today!” **Mrs. Smith:** “Oh yeah?” **PT:** “He will be so pleased!” **Mrs. Smith:** “Oh, okay, good!” (smiles) *Mrs. Smith may not know/trust the PT, but when she hears the PT knows Stan, she becomes a bit more relaxed.*	
**Patient Case #1 Part 3 (Acute Care Hospital): Empathic curiosity (Relationship building)** *Mrs. Smith is sitting upright. Her vitals are slightly elevated but stable. She continues to appear anxious.* **PT:** “You are doing well.” **Mrs. Smith** (anxious): “Yes. Well, I think …. Um.” **PT:** “It’s hard being in a strange place, isn’t it?” **Mrs. Smith:** “Yes …. It’s strange.” (smiles nervously) **PT:** “We are going to get you back home.” **PT:** “It will be nice to be home, won’t it?” **Mrs. Smith:** “Home.” (relaxes slightly) **PT:** “Thinking about home makes you relax.” (smiles) **Mrs. Smith:** “Yes.” (smiles more genuinely) **PT:** “Let’s take a walk, thinking about home …” .	
**Patient Case #2 (Community Based Rehabilitation Clinic): Reminiscence (Relationship building)** *Mary has a 5-year history of Alzheimer’s Disease with moderate dementia. She lives with her husband in the community. She recently fell and her physician recommended PT for balance training.* *She is reticent to engage in therapy. She is distracted and looking for her husband (who left to run an errand). The PT knows from her husband she is very proud of her long teaching career.* **PT:** “I understand you were a teacher for 30 years! Tell me what you loved about teaching …” . If open-ended questions are beyond Mary’s language abilities, then interactions can be phrased for more limited (yes/no) responses or simple acknowledgement: **PT:** “Did you enjoy teaching?” or “I bet the children loved you!” *To integrate into a therapeutic walking task (Motor Learning principle of task salience):* **PT:** “Let’s walk as though moving through rows of desks in a classroom,” or “Let’s pretend we are out at recess on the school grounds.”	
**Patient Case #3 Part 1 (In-Patient Rehabilitation Setting): Reality & Flexibility (Relationship)** *Mr. Jones is recovering from hip fracture surgery. He has moderate dementia and presents with some confusion.* *The PT may choose to help orient Mr. Jones to the reality of his situation.* **PT:** “Mr. Jones, you are in the hospital …. You fell and broke your hip …. Your recovery is going well.” *If Mr. Jones is asking for his sister Bess who died several years ago, the PT may respond to the perceived emotional source of the patient’s inquiry.* **PT:** “Are you missing your sister? Tell me about her,” *which may be a more gentle and productive response than the truth,* “Bess died several years ago.” *Whether an outright lie should be told* (“Bess will be back shortly”) *is controversial, but might be an option if Mr. Jones is perseverating on Bess and other options are failing*.	
**Patient Case #3 Part 2 (In-Patient Rehabilitation Setting): Errorless learning & Part-whole practice (Motor Learning)** *Mr. Jones is working on sit to stand from a chair to a walker. The PT identifies 3 components for safe sit to stand movement from a chair:* (1) *Scoot forward,* (2) *Push from chair to stand,* (3) *Hands to walker.* **PT:** “First, scoot forward … like this” (and demonstrates or facilitates). *The PT does not let errors occur, intervening with cues/handling* ***in anticipation*** *of errors.* *Mr. Jones goes through several practice trials with fading demonstration and fading verbal cues (same words, just fewer). Ultimately, the PT says:* “First?” *and Mr. Jones scoots forward.* **PT:** “Now, push from the chair to stand … like this” *(and demonstrates or facilitates). The PT does not let errors occur, intervening with cues/handling* ***in anticipation*** *of errors.* *Mr. Jones goes through several practice trials with fading demonstration and fading verbal cues (same words, just fewer). If there are adjustments required (*e.g.*, Mr. Jones needs to lean forward more for successful transition to stand), the PT facilitates the movement and may add a verbal cue.* *The PT may cluster practice of steps 2 and 3, every time the patient achieves full stand successfully (step 2), the PT prompts:* “Hands to the walker” *(step 3) to complete the skill.* *Within the PT session, the PT puts the components in context so Mr. Jones has an opportunity to practice the full sit to stand activity repeatedly.* *For optimal results, the entire care team must be consistent and united in the way they cue Mr. Jones for this task; thus, this becomes his default motor program for the activity over time.*	
**Patient Case #4 (In-Patient Rehabilitation Center) Behavior (Communication, Relationship)** *John had a bout with pneumonia and became very deconditioned in the acute care hospital. The nurse reports he was agitated during morning care and has refused to get in the wheelchair to go to his morning PT session. She describes his current status as “irritable.”* *The PT goes to John’s room to find him in his bed. His face and body seem tense and his manner gruff.* *The PT sits across from him, unrushed, with friendly face & body language.* **PT**: “Hi John. I’m _________, from PT.” **John**: “No, I’m not going.” **PT**: “Okay, that’s fine.” Pause. “We don’t need to go anywhere.” **PT**: “It must feel confusing to be here in the rehab center.” *Pause.* **PT:** “Everything is so unfamiliar.” *John is quiet but seems to be listening.* **PT:** “I want to help get you home safely with your daughter, Joanne, and your dog, Lola.” *Pause.* **PT:** “Shall we get you moving, so we can get you home?”	

Experienced PTs recognize the value of specialized training for working with ILwD [13, 15–18]. Those who work in geriatric residential settings may have more insights into the special needs of ILwD, but they still desire more information, particularly about late stage disease [15, 16]. PTs working in acute care settings, in- and out-patient rehabilitation, community-based, and home care environments may not have anticipated working with ILwD, but are more likely to see these patients as the population ages. In contrast to an older American study which found PTs’ knowledge of AD lacking, [19] a recent Canadian study [12] determined that PTs had knowledge about dementia, but needed more tools and confidence for clinical success, especially in managing advanced cognitive or behavioral issues. Qualitative studies of PTs working with ILwD consistently, regardless of country or practice setting, identify the need for more practical knowledge and targeted skills [6–8, 13, 15, 17, 18]. Attitudes about ILwD become increasingly negative as dementia severity increases, [16] and the term “dementia” often evokes an image of severe disease, not the community-dwelling individual with mild or moderate impairment. In a UK study, Bamford et al. [8] describe common categorization of ILwD as “not rehabable” and a tendency to attribute all patient problems to dementia, versus identifying potential contributing issues (e.g., pain, fatigue). An Australian study by Cations et al. [10] identified perceptions of incompatibility between “rehabilitation” and “dementia,” because of a default to a “palliative care” mindset. There is clear need for education to support an accurate, less fatalistic view of dementia, and empower PTs with skills and strategies to enhance rehabilitation efforts. Education related to working with ILwD has been associated with more positive attitudes [12] and more optimistic prognoses for outcome [17] among PTs.

The purpose of this paper is to introduce a framework for rehabilitation success with ILwD and to highlight how understanding the intricacies of these patients can inform and enhance clinical practice. Specific practical strategies, grounded in evidence as available, are divided into four primary components: (1) Establish a personal RELATIONSHIP, (2) Use intentional verbal and nonverbal COMMUNICATION, (3) Understand and optimize MOTOR LEARNING capabilities; and (4) Create a safe, purposeful ENVIRONMENT.

## The model

**Fig. 1:**
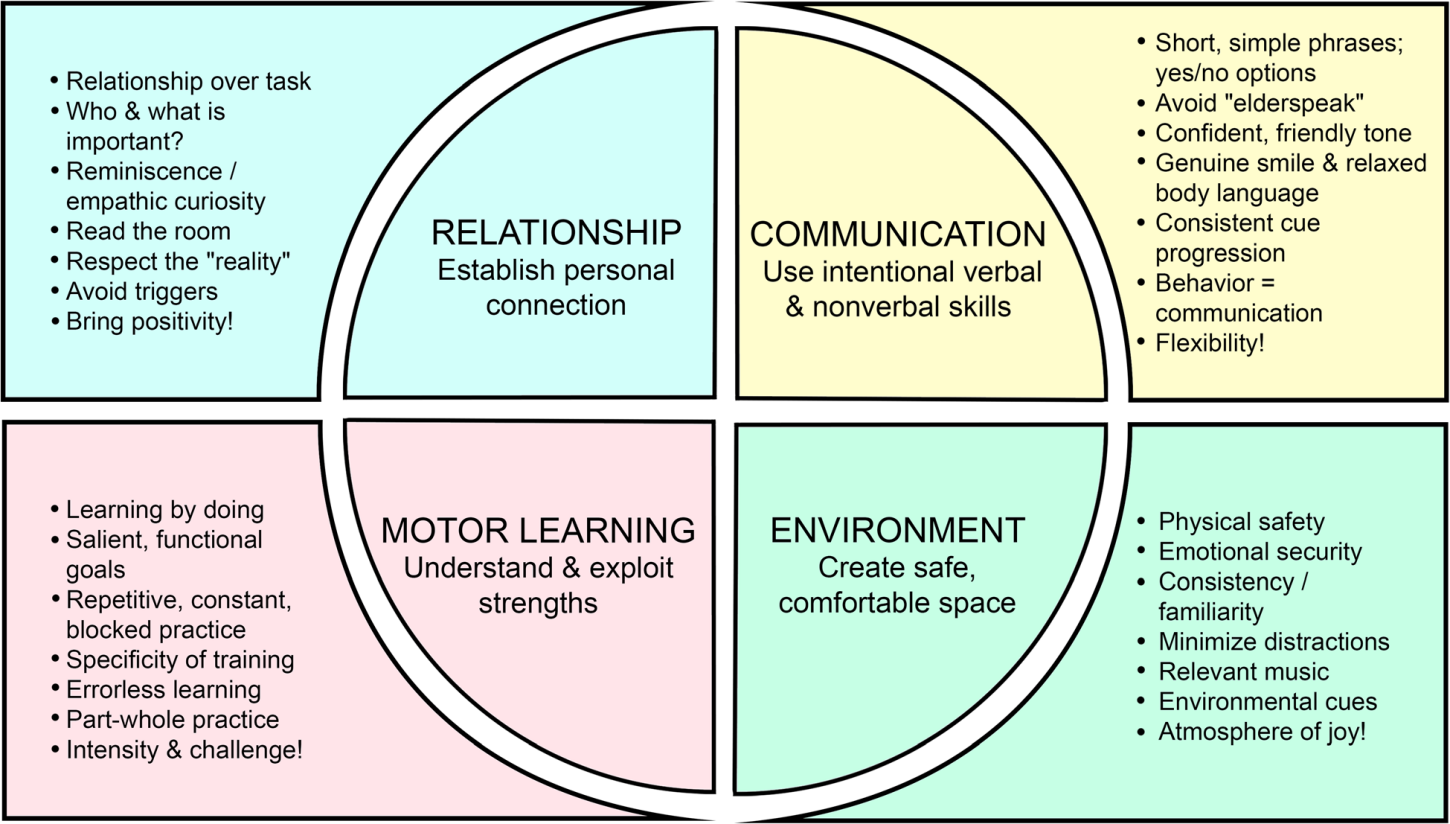
The model at a glance

This model (Fig. 1) is offered in response to the need for and benefit of education about working with ILwD. A narrative literature review exploring best practices in rehabilitation, nursing, and allied health professionals led to the four major elements of the model. These four areas are inherently involved in PTs’ interactions with all patients. PTs are movement experts who establish therapeutic relationships and tailor motor learning strategies to the needs of their patients and whose clinical successes routinely rely on modification of communication, interventions, and environments to facilitate optimal patient response. The unique needs of ILwD are considered in this model which provides practical and, in many cases, evidence-based recommendations for therapeutic success.

### The therapeutic RELATIONSHIP: establish a personal connection

Person-centeredness is a philosophy of care grounded in a genuine understanding of the individual [20]. In rehabilitation, Clare [21] frames person-centeredness in understanding the unique experiences, values, motivations, strengths, and needs of ILwD. The person, and the relationship with that person, are top priority. Personal information (from documentation or caregivers) can fuel relationship building; however, PTs also need to connect with patients when little or no personal data is available.

#### Know who and what is uniquely important

Knowing names of important people, places, and things, and having insights into relationships, professions, pastimes, and passions all help to create a picture of the patient. Making relevant conversation with personal references displays prioritization of relationship over task. Personalized interactions (versus generic pleasantries or focus on therapy) can make the person feel “seen” and “known,” important components of effective person-centered care [20, 22]. In the context of rehabilitation, PTs [8, 14, 15], ILwD, and their caregivers [23–25] recognize the value of this. Integrating the name of a loved one or a specific hobby in the context of goal setting can be heartening to a confused patient. Consider Patient Case #1 Part 2 (Table 1). As with all strategies presented here, the PT has to “read the room” to assess effect. If using personal information is met with paranoia, the PT needs to redirect conversation and reevaluate use of this tactic.

When there is no access to personal information, other strategies will support a therapeutic relationship. McGilton et al. [26] proposed that reliability (inclusive of trust, protection, and acceptance), empathy (with sensitivity to changing needs), and consistency (predictability) are pivotal to a caregiving relationship. Even in later stages of dementia, meaningful relationships can be established, evidenced by sharing of emotion, affection, and desire for future interaction [27]. PTs should enter relationships with expectations of success.

#### Reminiscence & empathic curiosity

Reminiscence Therapy (structured use of memories, experiences, and prompts) can have a positive impact on mood and communication/interaction, [28, 29] and sharing memories can allow healthcare providers to better see and know the person with dementia [30]. In rehab, integrating reminiscence concepts can foster the therapeutic relationship and direct functional activities. Consider Patient Case #2 (Table 1). If reminiscence strategies are elusive, the concept of “empathic curiosity” [31] allows interaction in the here and now. Using brief sentences, watching for and responding to emotional cues and metaphors, and sharing responsibility for the interaction can support the therapeutic relationship. Consider Patient Case #1 Part 3 (Table 1).

#### Recognize & accept the offered reality

There are differing opinions and no strong guidance from research [32, 33] about the value of letting a patient’s reality be THE reality in dementia care. Orientation to the reality of a situation may be appropriate, but quizzing or asking “Do you remember …? ” is rarely productive. Responding to the perceived emotional source of an individual’s statements or behaviors can be validating and helpful, and may be more gentle and productive than a hard to hear truth. Whether lying to a patient should be an option is a complex and controversial determination [34]. Consider Patient Case #3 Part 1 (Table 1). Being flexible is a sound strategy. Perhaps PTs should consider it a privilege to enter the offered reality and do so with grace and humility, rather than feel compelled to force someone back into a world that no longer comforts them.

#### Learn what brings joy and triggers negativity

Understanding a person’s “good life” helps the PT to frame interventions in an overtly meaningful way. Celebrating success and progress is motivating. Behavioral and psychological symptoms of dementia are impacted by patient, caregiver, and environmental factors [35]. Things that increase patient stress (e.g., acute medical issues, unmet needs [food, hydration, toileting, sleep], lack of meaningful activity, uncomfortable environment) or caregiver stress will have a negative effect on patient behaviors [35]. Saying/doing the “right” thing can endear the PT to a patient; saying/doing the “wrong” thing can bring a session to a screeching halt. ILwD may be surprisingly aware of a care provider’s demeanor, and if they perceive the PT as rushed or distracted (e.g., documenting while treating), this may trigger negative reactions.

### Use purposeful COMMUNICATION

Communication tips are abundant on every dementia association/society web page, but empirical research directing best practice is scarce, and very little is known about how ILwD experience communication [36]. Van Manen et al. [37] created a communication model from a scoping literature review, demonstrating the complexity of nursing staff-patient communication in dementia care, many components of which are represented here. Communication is intricately entangled with relationship building, and person-centered care requires a mindset of “working with” rather than “doing to,” [38] which requires the PT to be patient and humble. As with all older adults, assuring that ILwD are equipped with appropriate hearing and vision support will enhance communication efforts.

#### Verbal “Rules of Engagement”

Research related to communication between care providers and ILwD consistently concludes that use of short, simple phrasing and yes/no options can enhance interactions [39–41]. Other potentially useful strategies include: eliminating distractions, repetition and/or paraphrasing, reassurance, and patience (being comfortable with silence). PTs should be careful not to undermine patient capabilities by defaulting immediately to the most basic communication strategies, as those with mild to moderate dementia may have the ability to participate in interactive discussions, follow multi-step commands, or choose from several options.

Excessively slow speech by care providers does not facilitate communication, [39–41] and, in fact, “elderspeak” (also called “infantilizing,” where caregiver speaks slowly, with elevated pitch, and terms of endearment) can lead to decreased self-esteem and resistiveness to care [42]. More important than slowing of speech is purposeful pacing, allowing time for processing and response formulation. A confident, friendly voice with deliberate intonation should clearly indicate whether a statement or question is being offered. Attentively and actively listening allows the PT to follow the lead of the person with dementia and be a partner in communication.

#### Non-verbal “Rules of Engagement”

A measured approach, a genuine smile (sorely missed while hidden behind a mask during the Covid-19 pandemic!), friendly eye contact, and a relaxed demeanor are facilitators to communication. Positioning at eye level (i.e., sit if patient is sitting or supine) can equalize a perceived power dynamic. ILwD may have preserved sensitivity to non-verbal communication and emotional expression of others well into the disease, [41] even as language skills are failing, so sending positive non-verbal messages via face, body language, posture, and movement is essential. Excellent observation of patient non-verbals will help the PT pick up on physical or emotional discomfort that can interfere with therapy.

#### Recognize behavior as communication

Humans react to the world as they perceive it. Behavior must be evaluated in context, as odd behavior may have a simple explanation: A patient who removes her shirt in the PT gym feels hot! Uncertainty, discomfort, or fear can lead to cantankerous behavior. In post-hip fracture rehabilitation, McGilton et al. [43] studied allied health professionals’ perceptions of behaviors interfering with care in ILwD. Anxiety and irritability were most common, and strategies for management included being calm and reassuring, building relationship, and focusing on individual versus task, but few allied health staff prioritized assessing the reason for the behavior as a component of management [43]. Behavioral issues are common, but not an inevitable sequelae of dementia [44]. When faced with unexpected behaviors, PTs must problem solve! Pain or fear may masquerade as agitation; fatigue or confusion may cause disengagement. Before concluding a patient is “uncooperative” or “non-participatory,” the PT should work to get to the source of obstructive behaviors and try to manage the root problem. Consider Patient Case #4 (Table 1).

#### Mindful progression of cues

Communicating effectively requires cues to overcome deficits in attention, language, sequencing, and/or judgment [45]. An algorithm of progressive cuing strategies for ADLs introduced by Beck et al. [45] translates to an intuitive progression: Verbal Prompt → Model or Gesture → Physical Prompt → Physical Guidance → Physical Assistance. PTs must determine: [1] the ideal response time before repeating the cue [2]; the minimal amount of cuing that allows the patient to be successful within an activity, and [3] how cuing needs change with task, mood, or time of day. Cuing strategies are pivotal within motor learning and relearning context. In AD in particular, apraxia is not unusual, often presenting clinically as difficulty with imitating gestures, following demonstrations, or mimicking use of tools, [46, 47] so recognizing when these are and are not viable cuing strategies is important.

#### Be flexible

A toolbox of rapport building and communication strategies and flexibility within and between therapy sessions is advisable. Qualitative studies of PTs experienced in working with ILwD identify themes of “be on your toes” [48] and “think outside the box.” [14] Redirection to benign topics (e.g., the weather, the curtains) or go-to topics (e.g., favorite pet or sports team) may be useful for defusing a situation. Validation of how someone is feeling can create an alliance with an upset patient (e.g., “I know you’re mad. I wish we could start this day over,” “I see you’re upset, let me help”). Communicating to engage and connect supports an authentic therapeutic relationship.

### Understand & exploit MOTOR LEARNING strengths

Motor learning literature as it relates to ILwD is limited, but provides some important themes. Rehab focus may be on re-learning skills (e.g., sit to stand, activities of daily living) or may be on skills requiring new learning (e.g., novel use of an assistive device). These strategies can be useful in both contexts.

#### Prioritize procedural learning

A distinction between procedural/implicit and declarative/explicit memory and learning, and the neural substrates for different types of learning can help direct skill training [49–51]. Current motor learning researchers recognize a spectrum versus a dichotomy of implicit and explicit learning, and specific strategies may not be easily categorized as implicit or explicit [52]. For the purposes of this paper, procedural/implicit training is most succinctly understood as “learning by doing,” wherein a motor skill is acquired (or reacquired) using repetitive practice without intentional cognitive oversight. The cerebellum, basal ganglia, and sensorimotor cortical regions all play critical roles in procedural learning [49]. Declarative/explicit learning integrates cognitive strategies with motor practice, such as focused attention and awareness, verbally describing movement, reflecting on performance, and comparing outcome to previous performance. Medial temporal lobe function (hippocampus and adjacent structures) is highly implicated in explicit learning, [49] and these regions are well known to be involved early in AD, rendering these strategies less useful. Research directs prioritization of procedural learning strategies for ILwD, particularly those with AD, with whom this premise has been most studied [49, 50, 53–56]. Authors have been unable to draw conclusions about relationships between severity of dementia and procedural learning capabilities, [55, 57–59] meaning even individuals with moderate to severe dementia should have the opportunity to train motor-based functional tasks with purposeful procedural strategies. PTs should not summarily discount all declarative strategies, especially with individuals with mild dementia, [60, 61] but they must quickly assess usefulness (e.g., Asking someone to self-assess performance on an obstacle course might elicit a meaningful critique or undesirable anxiety).

#### Consider salience of tasks

Saliency is a relevant component of motor learning for all populations, [62] but even more so for ILwD [8]. Functional relevance may need to be more obvious for ILwD, where lower extremity strengthening is disguised as sit to stand activity drills and balance training is clearly framed within a motivating goal (e.g., “This will make it easier for you to feed your cat, Tabby”). Dutzi et al. [63] demonstrated the capability of rehab participants with mild-moderate dementia to accurately identify functional limitations and set meaningful goals using a structured approach. This reminds PTs not to make assumptions about patients’ insights into their own needs and represents an important integration of person-centered care.

#### Intentionally design practice sessions: repetitive, consistent, constant, & blocked

Classic work by Dick and colleagues [57–59, 64] and literature reviews [50, 53] have been influential in guiding practice structure. Definitions of terminology are included in Table 2. Intentional design of training sessions should include repetitive, consistent, constant (vs. variable), and blocked (vs. random) practice. Massed practice is generally desirable, but fatigue impacts learning, [49, 50] and distributed schedules may be preferred for some patients. Classic motor learning theory favors variable, random practice sessions, which aim to broaden motor programs, preparing learners for real world, unpredictable demands. This requires the learner to have the cognitive wherewithal to: [1] store and later retrieve performance data, [2] evaluate performance on different versions of tasks, and [3] move easily between tasks. ILwD, particularly those with AD, lack the ability to encode, store, and retrieve information. They lack the relational processing (required for variable practice) and the cognitive flexibility to move swiftly between tasks (required for random practice), making constant, blocked practice more effective [57–59, 64]. Little is known about optimal feedback type and schedules for ILwD, and processing feedback data may rely too heavily on cognition to be of real use [50]. Some studies suggest visual feedback may be important in motor learning, [50, 65] which highlights the need for appropriate, clean corrective lenses during therapy.

**Table 2:** Terminology for Motor Learning & Practice Schedules

Declarative / Explicit Learning	Integrating cognitive strategies with motor practice (e.g., attention to & awareness of movement, verbally describing / narrating movement, reflecting on / assessing movement, comparing to previous performance)
Repetitive Practice:	Multiple recurring trials of movement strategy
Consistent Practice:	Similar movement strategy from trial to trial
Constant Practice:	Similar task parameters from trial to trial (e.g., practice sit to stand from favorite chair)
Variable Practice:	Different task parameters from trial to trial (e.g., practice sit to stand from multiple chairs of varying surface, height, compliance, & stability)
Blocked Practice:	Cluster and complete trials for one task prior to moving to next task (e.g., complete transfer training before initiating gait training)
Random Practice:	Intermingle task activities within practice session (e.g., integrate mobility, transfer, and gait activities throughout session)
Massed Practice:	More practice than rest in a session
Distributed Practice:	More rest than practice in a session

#### Specificity of training

PTs should strive to create a therapy environment that closely mimics real life. ILwD who learn tasks through constant practice have rigid motor programs that are not easily modified, making specificity of training important for learning/relearning functional tasks [53, 59]. Therapy provided in the living environment (e.g., home care, residential care) has the benefit of being relevant and specific to daily life.

#### Errorless learning / spaced retrieval / part to whole practice

Errorless learning uses specific strategies (e.g., no guessing, stepwise approach, modeling, vanishing verbal/visual cues, spaced retrieval) [66] to minimize or eliminate errors during training. Avoiding patient-generated motor errors decreases the chance that a faulty movement becomes the default motor strategy. Consistency in error avoidance requires excellent observation and anticipation of movement and a commitment from the entire care team. Errorless learning has been demonstrated superior to “errorful”/trial and error learning for new, non-functional procedural tasks in ILwD [67, 68]. In the context of relearning Instrumental Activities of Daily Living (IADL), including technology use (e.g., phone, computer, appliances), errorless learning is a useful strategy, equivalent [60, 61, 69, 70] or superior [71] to trial and error learning. Spaced retrieval is a formulaic cue fading strategy within errorless learning. The PT increases intervals between cues with correct performance, and immediately corrects performance and decreases cue intervals upon anticipated errors. Spaced retrieval has been effectively used in IADL and functional training in ILwD [72, 73]. Part-whole practice, or deconstructing tasks into component parts, has been effectively used in IADL and sit-to-stand training [60, 74]. Forward chaining (adding next component part upon mastery of preceding step) provides the opportunity to practice parts in relation to one another. Within each treatment session, training culminates with whole task performance. Consider Patient Case #3 Part 2 (Table 1).

#### Sufficient intensity & challenge of training

A common misconception is that older adults will not tolerate intensive training. Monitoring physiological and cognitive/emotional response to therapy allows PTs to make informed decisions about increasing/decreasing level of intensity and challenge within therapy. Rest breaks should be offered judiciously, when needed, not out of habit between activities. Evidence consistently demonstrates high-intensity exercise is safe and effective for ILwD [75]. Physical activity is neuroprotective and supports neuroplasticity, specifically in brain regions implicated in dementias [76]. PTs can confidently and competently oversee intensive interventions and encourage patients to work hard! Sondell et al. [23] found that feeling challenged by exercises/activities was a positive and rewarding experience, associated with increased confidence and self-esteem in ILwD.

A long recognized enemy to functional independence is “excess disability,” in which ILwD are functionally more disabled than they should be, given their impairments [77]. This is often the result of diminished opportunity for task performance, driven by well-intentioned or time-sensitive caregiver assistance. Loss of opportunity leads to loss of skill. Recovery may be possible with new opportunity, [77, 78] which bodes well for rehabilitation and inspires deliberate education of caregivers to provide task opportunities, while being sensitive to their daily demands.

### Attend to ENVIRONMENTAL characteristics

Movement is the product of the person, the task, and the environment. Often, PTs have little environmental control, but being mindful of the therapeutic atmosphere can help to meet the needs of ILwD.

#### Prioritize patient safety and comfort

A safe, calm, and predictable environment is critical for ILwD, who value a sense of security in rehabilitation [23]. Patients desire physical and emotional safety, but sometimes busy staff prioritize physical safety at the expense of emotional safety and dignity [79]. Exceptional communication and relationship building strategies contribute to emotional safety and security when the physical environment is beyond the therapist’s control.

#### Attend to consistency and familiarity

When memory is impaired, a sense of routine can be reassuring; even without “remembering,” things may feel familiar and comfortable. Thus, consistency in place, people, and timing of therapy may enhance success. While this has not been empirically studied, experienced rehab professionals highlight the importance of consistency and familiarity within the therapeutic environment [10, 14].

#### Minimize distractions

Minimizing distractions for ILwD is intuitive. Beck et al. [45] identify “stimulus control” as the initial step in their cuing progression framework, for instance closing the door or repositioning within a room to face away from distracting activity. Noise reduction/regulation has been shown to decrease problematic behaviors in ILwD and has implications for rehabilitation [80, 81]. Any negative sensory experiences (e.g., foul odors, uncomfortable temperature) can potentially impact behavior [82] and impede rehab efforts and therefore should be managed.

#### Environment to support function / participation

Well-lit environments, natural light when possible, and avoidance of glare are anecdotally recommended. Given the prevalence of visual-spatial and spatial-cognitive impairment in this population, [83, 84] it may be difficult to tease out contributing constraints to motor learning, so enhancing environmental opportunity for success is important. Way-finding cues (e.g., signs/pictures for toilet) may be useful for functional independence, and distracting cues (e.g., camouflage door with mural) may help manage exit seeking behavior [85]. Relevant, patient-preferred ambient music can support positive behaviors [80] and can be easily integrated with rehab efforts.

#### Atmosphere of joy

While it may seem far-reaching, creating a positive atmosphere and capitalizing on the pleasure that recovery of movement and meaningful activities bring, goes a long way. Patients and families are clear that enjoyment is a priority in exercise and rehabilitation [8, 86, 87]. Being in the moment with a patient, having a laugh, making strides toward an agreed upon therapeutic goal are all cause for celebration!

## Discussion & conclusion

Dementia brings the slow demise of memory, function, and identity. In reorienting the focus from what is lost to what abilities remain and potential for gain, PTs create opportunity for rehabilitation success. This framework is intended to help PTs understand some idiosyncrasies of ILwD and exploit strengths and positive characteristics. A “strength-based approach” in dementia care has shown some success [88, 89] and is pivotal to person-centered care. Focusing on “reablement” and “living well” with dementia could reframe services and policies related to rehabilitation [21, 90]. Those with dementia are clear in their desire for others to focus on what they *can* do, not what they cannot [91, 92].

In post-hip fracture rehabilitation for ILwD, underutilization of rehabilitation and physical therapy services is common, despite evidence of benefit [93–95]. Physical rehabilitation eligibility and placement decisions are often made based upon limited exposure, in poorly suited environments, by staff under-trained in the management of this population, leading to underestimation of rehab potential and denial of future PT services [6, 14]. The framework offered here provides a grassroots effort to help enhance dementia care one PT at a time, while still working toward systemic solutions to this problem.

The model is intended to be dynamic and flexible, encouraging PTs to capitalize on parameters that are within their control at any given time. Various levels of dementia and different settings may render some strategies more or less available or effective than others. Relationship and communication are prioritized and interrelated, as communication serves relationship building. Personal information is useful, but when unavailable, excellent communication, conveying empathy and investment can foster connection. Intentional motor learning strategies and practice design should be directed toward salient functional goals, developed in partnership when possible. Environmental strategies may be limited (e.g. close door, turn off television), but recognizing the importance of creating a sense of security and comfort is invaluable. Helping family and other care providers understand some of these strategies can serve to support rehab efforts [8, 14, 15].

This model is offered as a starting point to bring attention to and encourage discussion about best practices for rehabilitation with ILwD. Clinicians who work regularly with ILwD may anecdotally support the model, but it has yet to be formally tested. Evidence is provided to support some, but not all components of the model and as more evidence is available within and beyond the four major sections, this could warrant modifications. This model is in response to the documented need for targeted knowledge and specific skills to support PTs in working with ILwD [6, 13, 15–18]. If an appreciation for these factors was among core competencies for PTs working with ILwD, perhaps there would be significantly fewer patients written off as “uncooperative” or “unable to participate” in PT.

## Data Availability

Not Applicable.
